# Undergraduate pharmacy students’ preference for case-based learning: a discrete choice experiment in China

**DOI:** 10.3389/fphar.2025.1529492

**Published:** 2025-02-28

**Authors:** Zhibo Guo, Yuxing He, Jianzhou Yan

**Affiliations:** ^1^ School of Basic Medicine and Clinical Pharmacy, China Pharmaceutical University, Nanjing, China; ^2^ School of International Pharmaceutical Business, China Pharmaceutical University, Nanjing, China

**Keywords:** case-based learning, discrete choice experiment, undergraduate pharmacy student, learning preference, pharmacy education

## Abstract

**Background:**

Understanding students’ preferences is crucial for developing targeted teaching strategies and improving educational outcomes. This study aimed to investigate Chinese undergraduate pharmacy students’ preferences for case-based learning (CBL).

**Methods:**

We conducted a discrete choice experiment (DCE) to quantify preferences for CBL. Six key attributes associated with CBL preferences were identified: case modality, provider type, group size, case authenticity, case complexity, and examination format. An online questionnaire was administered to undergraduate pharmacy students from two universities in China. The data were analyzed using a mixed logit model to estimate preference weights, assess the relative importance of the attributes, and predict uptake rates. Additionally, interaction effects and subgroup analysis were employed to identify heterogeneity in preferences among different student groups.

**Results:**

A total of 613 participants completed the questionnaire and 482 participants were included in the analysis. The most influential attribute was case modality, with scenario simulation strongly preferred over paper modality. Case authenticity and provider type were also significant factors, followed by group size and case complexity. Examination format did not significantly affect preferences. We found heterogeneity in preferences between different groups of students. The highest uptake was achieved when cases were presented in scenario simulations by clinical instructors in small groups, using real cases of low complexity alongside the traditional written examination.

**Conclusion:**

The study highlights the importance of case modality with scenario simulation, case authenticity, and provider by clinical instructors among Chinese undergraduate pharmacy students for CBL, and suggests the need for personalized CBL approaches to accommodate different preferences.

## 1 Introduction

Effectively addressing clinical challenges is an essential skill for pharmacy professionals involved in patient care and medication management. Pharmacy students, as future practitioners, must be equipped with a robust theoretical foundation and the practical skills to apply this knowledge to real-world clinical scenarios. The pharmacy education system in China is undergoing significant reforms to align the curriculum with the evolving demands of clinical practice (Hu et al., 2014). Teaching methods in Chinese pharmacy schools are diverse. While traditional lecture-based teaching remains prevalent, innovative approaches such as the flipped classroom and team-based learning are increasingly adopted, emphasizing student engagement and interaction. Practical training, such as clinical internships and simulations, is prioritized to develop students’ hands-on skills. Blended learning, which integrates online resources with face-to-face instruction, is widely implemented. Assessment methods have evolved to be more comprehensive, incorporating both traditional exams and practical skill evaluations to accurately assess students’ overall competencies.

Case-based learning (CBL) has emerged as an effective educational strategy that fosters the development of essential competencies by immersing students in simulated clinical contexts. This approach enhances their clinical thinking and decision-making skills (He et al., 2024). CBL allows students to apply and integrate their pharmaceutical knowledge within a safe learning environment, enabling them to tackle complex problems (Fasinu and Wilborn, 2024). Such preparation is crucial for equipping students with the skills necessary to address challenges they may encounter in future clinical practice. By engaging in realistic scenarios, students not only deepen their understanding of theoretical concepts but also cultivate the ability to think critically and respond effectively in real-world situations.

Although CBL has proven effective in enhancing the academic performance and analytical skills of medical and pharmacy students, developing effective CBL curricula remains a considerable challenge (Jacob et al., 2019). Several critical factors must be considered to maximize the effectiveness of CBL during the design phase. These factors include the mode of case presentation, the type of facilitators, the optimal group size for interactive learning, the realism of case studies to ensure relevance to real-world scenarios, the appropriate level of case complexity to challenge students, and the assessment techniques used to evaluate student understanding (Jacob et al., 2019; Yao et al., 2023).

Understanding student preferences is crucial for developing targeted teaching strategies and improving educational outcomes (Senko et al., 2012a). Previous research indicated that 84% of students at a medical college in India believed that CBL is a superior teaching-learning method compared to traditional didactic lectures (Kaur et al., 2020). Among medical students in Iran, the most significant motivational outcomes of CBL sessions included the applicability of basic sciences and the engaging nature of the sessions (Alizadeh et al., 2024). In China, the preferences of undergraduate nursing students for CBL programs were influenced by factors including the provider, case modality, group size, feedback, and case content (Yao et al., 2023). Studies from various countries indicated that while CBL was generally favored among students, the specific elements that contributed to its effectiveness varied depending on cultural and educational contexts. However, research on the preferences of undergraduate pharmacy students regarding CBL and the factors influencing these preferences is limited. The Discrete Choice Experiment (DCE) is a robust method for assessing individual preferences (Lancsar et al., 2017). In this approach, participants are given a selection of alternatives, each defined by specific attributes, and they are asked to choose their preferred option. Additionally, this method is particularly useful for predicting choice probabilities under different scenarios.

This study aims to explore the preferences of Chinese undergraduate pharmacy students for CBL and to dissect the factors that shape these preferences. The findings of this research will aid pharmacy educators in selecting and refining CBL to more effectively meet students’ learning needs and improve the quality of pharmacy education.

## 2 Methods

The DCE method was chosen for its ability to quantify trade-offs and preferences for attributes. The DCE for this study was developed following the current guidelines and recommendations (Bridges et al., 2011; Reed Johnson et al., 2013; Hauber et al., 2016). The key steps include defining the research question, identifying attributes and levels, constructing choice tasks, collecting data, and analyzing data.

### 2.1 Identifying attributes and levels

To identify the attributes and levels presented to participants, we conducted a comprehensive literature review. We identified 13 candidate attributes from a DCE on CBL conducted among nursing students (Yao et al., 2023) and from interview studies on pharmacy CBL (Jacob et al., 2019; Kaur et al., 2020; Tsekhmister, 2023), after consolidating similar attributes and eliminating irrelevant ones (Supplementary Table S1). Subsequently, a focus group discussion was conducted with 15 undergraduate pharmacy students at China Pharmaceutical University. To increase the diversity and representativeness of participants, we distributed recruitment information through multiple channels, such as classroom announcements and social media. The selected participants included students of different genders, ages, and academic performance, which provided us with a broader perspective. In the discussion, participants were encouraged to share their views on the candidate attributes. After confirming that the candidate attributes were appropriate and did not require any additions or deletions, participants ranked the 13 attributes in order of importance, and the results were validated to ensure alignment with their opinions. Additionally, the levels were developed based on feedback from the literature survey and the focus group discussion. Ultimately, we identified six attributes and their levels that were closely associated with an individual’s propensity to engage in CBL (Table 1).

**TABLE 1:** Attributes and levels in the discrete choice experiment.

Attribute	Level	Explanation
Case modality	Paper	Cases are provided in text form
	Scenario simulation	Cases are presented in a role-playing format, where students act as patients/pharmacists/physicians
	Video	Cases are provided in video format
Provider type	Clinical instructors	Taught by experienced clinical pharmacists
	Academic experts	Taught by university faculty
	Mixed	Taught by both clinical instructors and academic experts
Group size	Large	14–16 participants
	Medium	9–11 participants
	Small	4–6 participants
Case authenticity	Real	Actual patient cases derived from clinical practice
	Virtual	Virtual cases designed according to educational needs
Case complexity	Low	Simple cases suitable for foundational learning
	Medium	Moderate difficulty that challenges application of knowledge
	High	Complex cases requiring advanced analysis and critical thinking
Examination	Traditional written exam	Assesses knowledge through written tests
	Oral presentation	Students are required to present on a specific topic

### 2.2 Experiment design

The questionnaire consisted of two sections, namely, demographic characteristics and DCE. The former included age, gender, year level, rank, clinical internship experience, and plan after graduation, while the latter comprised an explanation of the attributes and levels and choice sets. Each choice set presented two side-by-side scenarios, which described a hypothetical CBL characterized by six different attribute parameters. An opt-out option was included, indicating that neither CBL program was preferred. An example of choice set was shown in Table 2.

**TABLE 2:** Choice set example.

	Program A	Program B
Case Modality	Scenario simulation	Paper
Provider type	Academic experts	Clinical instructors
Group Size	Small	Large
Case authenticity	Real	Virtual
Case complexity	Low	High
Examination	Oral presentation	Traditional written exam

Which program do you prefer? □Program A □Program B □Neither.

The D-efficiency design was developed to generate 22 choice sets using Ngene software version 1.3.0 (ChoiceMetrics, Sydney, NSW, Australia), which were further divided into two blocks to reduce participant cognitive load. The D-efficiency design was terminated when further iterations did not significantly improve the design efficiency, that is, when the D-error stopped decreasing. The mean D-error of the design reported by the Ngene software was 0.361. We employed a D-efficiency design to maximize the information obtained, acknowledging that achieving an equal occurrence of all attribute levels across all 22 selection sets is impractical and could potentially introduce a certain level of bias into the study. The second choice was duplicated in each block and presented as the 12th choice set to check response consistency. Only the responses to the first eleven questions were used for the data analysis. Each participant was randomly assigned one block and asked to answer 11 choice sets. The order of the attributes in the choice sets was randomly presented to the participants.

A face-to-face pilot test was conducted among 30 participants in July 2024 before launching the formal survey. The pilot study enabled the preemptive identification and resolution of potential confusion among participants. No significant changes were made following the pilot test, as the feedback received indicated that the survey was largely comprehensible.

### 2.3 Survey

The sample size was determined using the rule of thumb proposed by Johnson and Orme [N > 500 × c/(t × a)] (De Bekker-Grob et al., 2015), which required at least 69 participants (with a maximum of three levels, eleven choice tasks, and two alternatives). Our goal was to collect 600 questionnaires to ensure the exclusion of unqualified questionnaires and enhance the reliability of the findings. An online survey was conducted between August and September 2024 to administer the questionnaires. Participants were recruited from two universities, namely, China Pharmaceutical University and Nanjing Medical University. Eligible participants were undergraduate students majoring in clinical pharmacy. The electronic survey disseminated via WeChat, a widely used messaging platform in China, allowing for easy access and participation. We recruited participants by sending the survey link in the WeChat groups of clinical pharmacy classes across various grades. Reminders were sent out at regular intervals to encourage participation until the target sample size was reached. Participation in the survey was voluntary, and informed consent was obtained from all participants prior to the survey. The consent form explained the purpose of the study, the confidentiality of their responses, and their right to withdraw from the study at any time without consequence. We were also available in the WeChat groups to promptly address any inquiries that participants had while they were filling out the questionnaire, ensuring a smooth response process. To ensure integrity, all questions must be answered before the survey can be submitted.

The exclusion criteria included participants who completed the survey in less than 1 min (which was one-third of the median time in the pilot survey), those who consistently selected the same answer for each choice (either consistently choosing the left or right option), and those who provided inconsistent responses in repeated choice sets. This study obtained ethical approval from the China Pharmaceutical University.

### 2.4 Statistical analysis

A mixed logit model was employed to estimate preference weights and relative importance (RI) of various attributes. An alternative specific constant (ASC) was included to indicate the preference for opt-out option. Dummy coding was used for all attributes. Random parameters were estimated using 500 standard Halton sequences, which achieved a stable and robust estimation. The sign of the coefficients indicates whether participants valued an attribute positively or negatively. The RI for each attribute was calculated by dividing the difference between the coefficients for the best and worst levels by the sum of all the attribute differences (Lancsar et al., 2007).

To analyze the heterogeneity of preferences, we constructed models containing interaction terms between individual-specific characteristics and attribute levels. The individual-specific characteristics included gender, year level, ranking, clinical internship experience, and plan after graduation. The positive coefficients of the interaction terms suggest that subgroups with specific characteristics placed greater importance on the attributes than those without such characteristics. Conversely, the negative coefficients of the interaction terms imply that subgroups with specific characteristics assigned less importance to the attributes compared to their counterparts.

Furthermore, the uptake rates of CBL were estimated under different scenarios using findings from the mixed logit model with main effects. The base-case scenario was characterized by the following attribute levels: paper case modality, provider by academic experts, large group size, virtual case, high case complexity, and oral presentation examination. Statistical significance was determined at P < 0.05 (two-sided), and 95% confidence intervals (CI) were calculated using the bootstrap method. Data analyses were performed using Stata 16 (StataCorp LLC, College Station, TX, United States).

## 3 Results

### 3.1 Participants characteristics

A total of 613 participants completed the questionnaire. After applying the exclusion criteria, 45 participants were excluded for completing the survey in under 1 min, 34 for consistently choosing the same option, and 52 for inconsistent responses across repeated choice sets. This resulted in an analytic dataset of 482 participants with a mean age of 19.47 years (Table 3). Among the participants, 62.46% were female, 64.73% were in their first or second year, and 36.10% ranked in the top 25%. Clinical internship experience was reported by 27.59% of the participants. Additionally, 28.84% of participants intended to work in a hospital, while 61.20% aimed to pursue further studies.

**TABLE 3:** Participants’ characteristics (n = 482).

Characteristic	Number (%)
Gender	
Male	181 (37.55)
Female	301 (62.45)
Age (years), mean ± SD	19.47 ± 1.55
Year level	
First to second year	312 (64.73)
Third to fifth year	170 (35.27)
Ranking	
Top 25%	174 (36.10)
25%–50%	166 (34.44)
50%–75%	87 (18.05)
Bottom 25%	55 (11.41)
Clinical internship experience	
Yes	133 (27.59)
No	349 (72.41)
Plan after graduation	
Work in a hospital	139 (28.84)
Non-hospital work	35 (7.26)
Continue further studies	295 (61.20)
Other	13 (2.70)
University	
China Pharmaceutical University	330 (68.46)
Nanjing Medical University	152 (31.54)

### 3.2 Preferences estimated using mixed logit model

The preference weights estimated using the mixed logit model were presented in Table 4. The results indicated that five of the six attributes significantly influenced preferences for CBL, with the exception of the examination method. Participants preferred the scenario simulation modality over the paper modality (P < 0.001). Additionally, clinical instructors were favored over academic experts as providers (P < 0.001). A small group size was preferred compared to a large group size (P = 0.015), and real cases were more favored than virtual cases (P < 0.001). Furthermore, low case complexity was preferred over high complexity (P = 0.006). However, traditional written exams and oral presentations did not significantly affect CBL preferences (P = 0.580).

**TABLE 4:** Preference estimated by mixed logit model.

Attribute and level	Coefficient (95% CI)	P-value	SD (95% CI)	SD P-value
Case modality (ref: paper)				
Scenario simulation	0.57 (0.44, 0.70)	<0.001	0.63 (0.43, 0.83)	<0.001
Video	0.18 (0.03, 0.34)	0.018	0.49 (0.27, 0.71)	<0.001
Provider type (ref: clinical instructors)				
Academic experts	−0.37 (−0.53, −0.21)	<0.001	0.61 (0.43, 0.79)	<0.001
Mixed	−0.14 (−0.28, 0.00)	0.055	0.19 (−0.19, 0.57)	0.327
Group size (ref: large)				
Medium	0.10 (−0.04, 0.24)	0.145	0.00 (−0.26, 0.25)	0.970
Small	0.23 (0.05, 0.42)	0.015	0.31 (0.08, 0.54)	0.008
Case authenticity (ref: real)				
Virtual	−0.43 (−0.55, −0.32)	<0.001	0.79 (0.65, 0.93)	<0.001
Case complexity (ref: low)				
Medium	−0.10 (−0.21, 0.02)	0.112	0.36 (0.12, 0.60)	0.003
High	−0.17 (−0.29, −0.05)	0.006	0.58 (0.39, 0.77)	<0.001
Examination (ref: traditional written exam)				
Oral presentation	−0.05 (−0.21, 0.12)	0.580	1.57 (1.38, 1.76)	<0.001
ASC	−4.45 (−5.16, −3.73)	<0.001	4.53 (3.87, 5.20)	<0.001
Model specification				
Log likelihood	−3912.70			
AIC	7869.39			
BIC	8038.23			

Abbreviations: ASC, alternative-specific constant; AIC, akaike information criterion; BIC, bayesian information criterion.

The coefficient for opting out was found to be −4.45 (P < 0.001), indicating that opting out negatively impacted participants’ utility, making participants more likely to choose CBL. Furthermore, most estimated standard deviations were significant, suggesting the presence of preference heterogeneity among participants.

**FIGURE 1:**
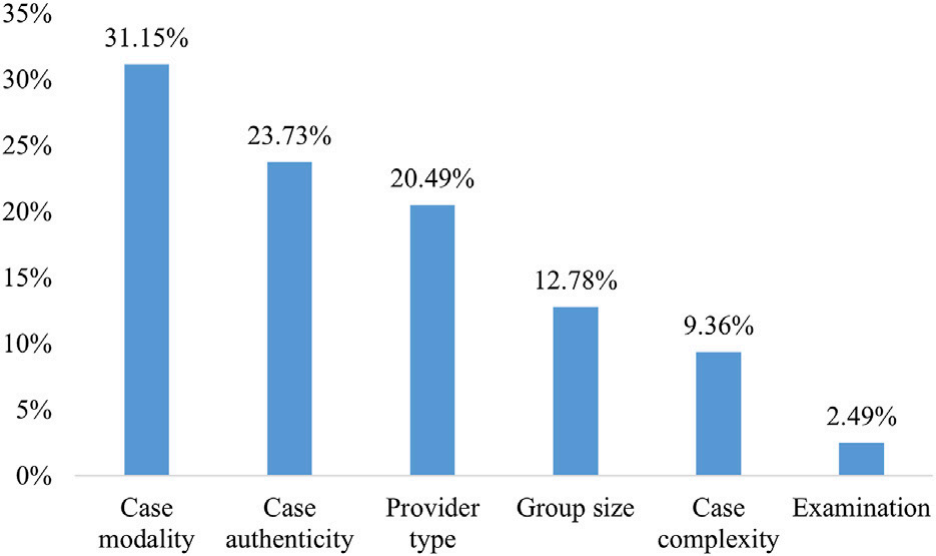
Relative importance of attributes in case-based learning preferences.

The RI results for six attributes (Figure 1) indicated that the participants assigned the highest value to the case modality (RI = 31.15%), followed by case authenticity (RI = 23.73%), provider type (RI = 20.49%), group size (RI = 12.78%), and case complexity (RI = 9.36%). The examination method was considered less important, with RI values of 2.49%.

### 3.3 Interaction effect

Six significant interaction terms were identified, as shown in Table 5. Males were more likely than females to choose cases with a higher level of complexity (β = 0.25, P = 0.035). Lower year students rated the case modality of scenario simulations as more important than higher year students (β = −0.28, P = 0.027). Students who ranked higher preferred scenario simulations (β = −0.14, P = 0.028) and video cases (β = −0.15, P = 0.014) over those who ranked lower. Students with internship experience preferred CBL more than those without internship experience (β = −1.99, P = 0.003), and they exhibited a more pronounced negative preference for high-complexity cases (β = −0.28, P = 0.035).

**TABLE 5:** Preference estimated by mixed logit model with main effects and interactions.

Attribute and level	Coefficient (95% CI)	P-value
Case modality (ref: paper)		
Scenario simulation	1.25 (0.78, 1.71)	<0.001
Video	0.89 (0.44, 1.34)	<0.001
Provider type (ref: clinical instructors)		
Academic experts	−0.37 (−0.53, −0.21)	<0.001
Mixed	−0.13 (−0.28, 0.01)	0.066
Group size (ref: large)		
Medium	0.10 (−0.04, 0.24)	0.143
Small	0.23 (0.05, 0.42)	0.019
Case authenticity (ref: real)		
Virtual	−0.43 (−0.55, −0.32)	<0.001
Case complexity (ref: low)		
Medium	−0.10 (−0.22, 0.02)	0.102
High	−0.32 (−0.54, −0.11)	0.003
Examination (ref: traditional written exam)		
Oral presentation	−0.03 (−0.19, 0.13)	0.705
ASC	−4.25 (−5.00, −3.50)	<0.001
Interaction terms		
Male* complexity high	0.25 (0.02, 0.49)	0.035
Year level* modality video	−0.28 (−0.53, −0.03)	0.027
Ranking* modality simulation	−0.14 (−0.26, −0.01)	0.028
Ranking* modality video	−0.15 (−0.27, −0.03)	0.014
Internship* ASC	−1.99 (−3.30, −0.67)	0.003
Internship* complexity high	−0.28 (−0.53, −0.02)	0.035
Model specification		
Log likelihood	−3892.98	
AIC	7847.96	
BIC	8085.87	

Abbreviations: ASC, alternative-specific constant; AIC, akaike information criterion; BIC, bayesian information criterion.

### 3.4 Subgroup analysis

Divergent preference patterns were observed within subgroups for the RI of different attributes (Supplementary Figure S1). Males placed the greatest emphasis on provider type (RI = 28.07%), while females prioritized case modality (RI = 35.37%). Among higher-year level students, the importance of examination method was notably high (RI = 33.05%), with a preference for oral presentations over traditional written exams. Students in the top 50% assigned a relatively low RI of 6.04% to case complexity, in contrast to those in the bottom 50%, who rated it as high as 16.70%, suggesting a preference for less complex cases. Students without clinical internship experience rated the RI for case modality at 34.77%, whereas those with such experience rated it lower, at 15.66%. Students with internship experience showed a greater emphasis on both provider type and case complexity.

### 3.5 Predicted uptake rate

The predicted uptake rates under different scenarios were depicted in Table 6. Initially, we adjusted one attribute at a time compared to the base case scenario. The highest uptake rate (27.75%) was achieved by changing the case modality from paper to scenario simulation, followed by changing the virtual case to real case, which resulted in an uptake rate of 21.37%. Similarly, changing the provider from academic experts to clinical instructors resulted in an uptake rate of 18.52%. Furthermore, the combination of several attributes significantly increased uptake rates. For example, when cases were presented through scenario simulations provided by clinical instructors, the uptake rate increased by 44.01% compared to the base case scenario. The uptake rate was as high as 71.24% when cases were presented using scenario simulation delivered by clinical instructors, in small groups, with real cases of low complexity, and with a traditional written exam.

**TABLE 6:** Predicted uptake rates under various scenarios.

Case modality	Provider type	Group size	Case authenticity	Case complexity	Examination	Uptake rate
Scenario simulation	Academic experts	Large	Virtual	High	Oral presentation	27.75%
Paper	Clinical instructors	Large	Virtual	High	Oral presentation	18.52%
Paper	Academic experts	Small	Virtual	High	Oral presentation	11.64%
Paper	Academic experts	Large	Real	High	Oral presentation	21.37%
Paper	Academic experts	Large	Virtual	Low	Oral presentation	8.54%
Scenario simulation	Clinical instructors	Large	Virtual	High	Oral presentation	44.01%
Scenario simulation	Clinical instructors	Small	Virtual	High	Oral presentation	52.94%
Scenario simulation	Clinical instructors	Small	Real	High	Oral presentation	66.76%
Scenario simulation	Clinical instructors	Small	Real	Low	Oral presentation	71.24%
Scenario simulation	Clinical instructors	Small	Real	Low	Traditional written exam	72.34%

### 3.6 Sensitivity analysis

The sensitivity analysis utilized data from the full sample of 613 respondents, including questionnaires that were excluded from the main analysis. Results obtained from a mixed logit model were presented in Supplementary Table S2, and the RIs of six attributes were shown in Supplementary Figure S2. Similar to the main analysis, case modality was the most valued attribute (RI = 32.44%), and the importance rankings remained unchanged.

## 4 Discussion

The study provided insight into the preferences of Chinese undergraduate pharmacy students regarding CBL. Our findings identified key attributes that significantly influenced students’ preferences for CBL. The most influential attribute was case modality, with scenario simulation strongly preferred over paper format. Next, case authenticity and provider type emerged as important factors. In addition, our study revealed heterogeneity in CBL preferences among different students. Furthermore, the uptake rate was highest when cases were presented using scenario simulation delivered by clinical instructors, in small groups, with real cases of low complexity, and with traditional written examination.

Our results indicated that the attribute with the highest RI value was case modality, with pharmacy students showing a clear preference for scenario simulations over paper-based case modalities. Scenario simulations offer a more interactive and immersive learning experience, which aids in the comprehension and retention of complex knowledge. In contrast to our findings, undergraduate nursing students showed a preference for video cases over paper and simulated cases (Yao et al., 2023). Such discrepancies may be due to differences in the populations studied.

Next in importance was case authenticity, as students significantly preferred real cases over virtual cases. This finding suggests that students highly value the relevance and applicability of learning materials to real clinical scenarios. Although virtual cases provide flexibility, they may result in a less immersive learning experience, potentially leaving students unprepared for the complexities of real-world clinical practice. This distinction is particularly important in medical education, where the stakes are high and the ability to apply knowledge in real-world situations is essential (Alizadeh et al., 2024).

The provider type attribute was identified as the third most important factor, with clinical instructors preferred over academic experts. This preference likely stems from students’ desire for practical insights and expertise that can only be provided by those with direct clinical experience. Clinical instructors offer a perspective that is more aligned with the realities of patient care, which is invaluable for students as they prepare for their future careers. The emphasis on clinical instructors aligns with findings that suggest students value instructors who can provide practical applications of theoretical concepts (Senko et al., 2012b).

The preference for smaller group sizes of 4 to 6 students suggests that participants appreciate personalized learning experiences that offer greater individualized attention and interaction. Smaller groups foster a collaborative learning environment, allowing students to more effectively participate in discussions, ask questions, and receive feedback. Consistent with our findings, previous research has shown that smaller groups can increase student engagement and participation, leading to improved academic performance and more positive attitudes toward collaborative learning (Kalaian and Kasim, 2017).

Although case complexity was less influential than other attributes, it still held significance for students. They appeared to prefer cases with a lower level of complexity, likely because simpler cases allow for more focused exploration of key concepts and skills. This is particularly beneficial for undergraduate pharmacy students who are still developing their foundational knowledge and clinical reasoning abilities. Consistent with our study, a previous study showed that novice learners often benefit from simpler cases that allow them to build foundational knowledge and skills before tackling more complex scenarios (Saravanan and Menold, 2022).

Contrary to expectations, examination methods did not have a significant impact on students’ preferences for CBL. This suggests that the method of assessment is not a primary driver of students’ preferences for CBL; rather, other aspects of the learning process and the development of clinical skills play a more crucial role in shaping their attitudes towards CBL.

Our study also revealed heterogeneity in preferences for CBL among different student groups. Students of varying genders, year levels, academic rankings, and clinical internship experiences exhibited diverse preferences for CBL, which was consistent with findings from previous research highlighting the influence of demographic factors on learning preferences (Aldosari et al., 2018; Yue and Lu, 2022). Therefore, it is critical for educators to consider these diverse factors when designing and implementing CBL programs to ensure that they meet the diverse needs of all student populations. For example, for lower-year students, we recommend CBL with incremental complexity. For those with clinical experience, we suggest using real-world cases and advanced simulations. These strategies can accommodate the diverse needs of different student groups.

Furthermore, we predicted the uptake rates of CBL under different scenarios, providing valuable insight into the potential effectiveness of various CBL configurations. This predictive analysis is crucial for understanding how different elements of CBL can be optimized to enhance student engagement. When adjusting only one attribute, the highest uptake rate was observed when the case modality shifted from paper-based to scenario simulation, emphasizing the significance of interactive learning environments. The highest uptake rate was achieved when cases were presented through scenario simulation by clinical instructors in small groups, using real-world, low-complexity cases alongside the traditional written exam. This finding suggests that the attractiveness of CBL can be effectively enhanced through comprehensive consideration of multiple attributes.

There were several limitations to this study. Firstly, the sample, although sizable, is drawn from only two universities, which may limit the generalizability of the findings to other educational contexts or regions within China. Secondly, the attributes considered in the DCE, while comprehensive, may not cover all aspects that could influence student preferences for CBL. Lastly, while the DCE is a robust method for assessing preferences, it may not fully capture the complexity of real-world decision-making processes.

## 5 Conclusion

This study indicated that Chinese undergraduate pharmacy students’ preference for CBL was influenced by factors including the case modality, case authenticity, provider type, group size, and case complexity. The findings also suggest the need for personalized CBL approaches to accommodate the diverse preferences of student subgroups. Educators can adjust these attributes to better meet the needs and preferences of undergraduate pharmacy students.

## Data Availability

The raw data supporting the conclusions of this article will be made available by the authors, without undue reservation.
