# Supplementary material for: Undergraduate pharmacy students’ preference for case-based learning: a discrete choice experiment in China
Source: Front Pharmacol. 2025 Feb 28;16:1529492. doi: 10.3389/fphar.2025.1529492 (PMC11906709; doi:10.3389/fphar.2025.1529492)
Supplement: Supplementary file 1 [file DataSheet1.docx]

Supplementary Material

**Table S1** Candidate attributes from literature

|  | Candidate attributes |
| --- | --- |
| 1 | Case content comprehensiveness |
| 2 | Case delivery form |
| 3 | Case modality |
| 4 | Provider |
| 5 | Group size |
| 6 | Feedback |
| 7 | Case authenticity |
| 8 | Case complexity |
| 9 | Interactivity |
| 10 | Diversity of cases |
| 11 | Resource support |
| 12 | Examination |
| 13 | Flexibility |

(a) Grouped by gender

(b) Grouped by year level

(c) Grouped by ranking

(d) Grouped by clinical internship experience

(e) Grouped by plan after graduation

**Figure S1** Relative importance of six attributes for each subgroup.

**Table S2** Preference estimated by mixed logit model in full sample.

| **Attribute and level** | **Coefficient (95% CI)** | **P-value** | **SD (95% CI)** | **SD P-value** |
| --- | --- | --- | --- | --- |
| Case modality (ref: paper) | | | | |
| Scenario simulation | 0.55 (0.43, 0.68) | < 0.001 | -0.64 (-0.83, -0.45) | < 0.001 |
| Video | 0.18 (0.03, 0.32) | 0.020 | -0.49 (-0.69, -0.28) | < 0.001 |
| Provider type (ref: clinical instructors) | | | | |
| Academic experts | -0.37 (-0.52, -0.21) | < 0.001 | 0.6 (0.44, 0.76) | < 0.001 |
| Mixed | -0.13 (-0.26, 0.01) | 0.070 | -0.22 (-0.5, 0.06) | 0.125 |
| Group size (ref: large) | | | | |
| Medium | 0.10 (-0.04, 0.23) | 0.154 | -0.01 (-0.23, 0.21) | 0.912 |
| Small | 0.20 (0.02, 0.38) | 0.033 | 0.19 (-0.17, 0.55) | 0.291 |
| Case authenticity (ref: real) | | | | |
| Virtual | -0.40 (-0.51, -0.29) | < 0.001 | 0.79 (0.66, 0.92) | < 0.001 |
| Case complexity (ref: low) | | | | |
| Medium | -0.09 (-0.21, 0.02) | 0.114 | 0.40 (0.19, 0.60) | < 0.001 |
| High | -0.18 (-0.30, -0.06) | 0.002 | -0.51 (-0.70, -0.32) | < 0.001 |
| Examination (ref: traditional written exam) | | | | |
| Oral presentation | -0.01 (-0.16, 0.15) | 0.923 | 1.51 (1.34, 1.68) | < 0.001 |
| ASC | -4.87 (-5.68, -4.07) | < 0.001 | 4.72 (4.07, 5.38) | < 0.001 |

Abbreviations: ASC, alternative-specific constant.

**Figure S2** Relative importance of six attributes in full sample.
